# Multimodal neuroimaging and AI integration in cognitive disorders: advances, challenges, and future directions for precision medicine

**DOI:** 10.1093/psyrad/kkag007

**Published:** 2026-03-11

**Authors:** Mingxi Dang, Bing Liu, Yaojing Chen, Zhanjun Zhang

**Affiliations:** State Key Laboratory of Cognitive Neuroscience and Learning, Beijing Normal University, Beijing 100875, China; School of Systems Science, Beijing Normal University, Beijing 100875, China; BABRI Centre, Beijing Normal University, Beijing 100875, China; State Key Laboratory of Cognitive Neuroscience and Learning, Beijing Normal University, Beijing 100875, China; State Key Laboratory of Cognitive Neuroscience and Learning, Beijing Normal University, Beijing 100875, China; BABRI Centre, Beijing Normal University, Beijing 100875, China; State Key Laboratory of Cognitive Neuroscience and Learning, Beijing Normal University, Beijing 100875, China; BABRI Centre, Beijing Normal University, Beijing 100875, China; Innovation Institute of Integrated Traditional Chinese and Western Medicine, Shandong First Medical University & Shandong Academy of Medical Sciences, Jinan, Shandong 250117, China

**Keywords:** multimodal neuroimaging, artificial intelligence, cognitive disorders, precision medicine, explainable AI, differential diagnosis, biomarker discovery, alzheimer’s disease

## Abstract

Cognitive disorders, with dementia as a primary exemplar, present profound diagnostic and therapeutic challenges due to their complex pathologies and heterogeneous presentations. Artificial intelligence (AI), particularly when applied to multimodal neuroimaging and clinical data, offers a powerful approach to advancing precision medicine in this domain. This comprehensive review first examines foundational AI algorithms, including artificial neural networks for feature extraction, multimodal fusion strategies (e.g. early, intermediate, and late fusion) for data integration, and explainable AI (XAI) techniques to enhance clinical transparency. The core focus is on the application of these multimodal AI frameworks across the dementia care continuum, encompassing improved differential diagnosis, early detection through presymptomatic biomarkers, development of predictive models for disease progression, and optimization of patient stratification for clinical trials. Despite significant advances, persistent challenges remain, including limited generalizability across populations and protocols, data scarcity for non-Alzheimer’s dementias and prodromal stages—exacerbated by demographic biases—and barriers to interpretability. We discuss solutions such as federated learning for privacy-preserving data sharing and advanced XAI techniques. Finally, we outline pivotal future directions, including intelligent sensor fusion for discovering novel early biomarkers, hybrid AI architectures combining generative and discriminative models, innovations for handling missing modalities, and robust multicenter data integration frameworks. By synthesizing these advances, this review highlights the role of multimodal AI in advancing precise diagnosis, early prediction, and therapeutic development for neurodegenerative and vascular cognitive disorders, while identifying key translational challenges for precision medicine.

## Introduction

Cognitive disorders, characterized by the progressive deterioration of cognitive and mental processes—including memory, executive function, and perceptual-motor abilities—pose a critical global health challenge in aging populations, with dementia being the most prevalent and severe clinical manifestation (Hugo and Ganguli, 2014). Neuroimaging elucidates intricate neuropathological substrates, revealing convergent mechanisms and disease-specific alterations that drive substantial symptom overlap and clinical heterogeneity (Serena *et al*., 2021). This pathophysiological complexity, compounded by dementia’s insidious preclinical onset, creates formidable barriers to early detection and precise diagnosis (Paraskevaidi *et al*., 2018; Ryan *et al*., 2018). Within this framework, artificial intelligence (AI) integrated with multimodal neuroimaging presents transformative potential for detecting early biomarkers and enabling personalized therapeutic strategies.

To accurately model the complex spectrum of dementia, AI requires data that capture the full pathological continuum, placing multimodal neuroimaging at the forefront of diagnostic innovation (Kumar *et al*., 2024). Traditional unimodal approaches offer fragmented perspectives: structural magnetic resonance imaging (MRI) quantifies cerebral atrophy yet overlooks functional compensation, while fluorodeoxyglucose positron emission tomography (FDG-PET) metabolic patterns show limited specificity—exemplified by Alzheimer’s disease (AD)-indistinguishable hypometabolism in 29% of dementia with Lewy bodies (DLB) and 35% of frontotemporal dementia (FTD) cases (Mosconi *et al*., 2008). These limitations impede comprehensive pathogenesis elucidation and differential diagnosis, particularly in early or atypical presentations (Womack *et al*., 2011; Villemagne *et al*., 2015). Through multimodal integration, AI enhances diagnostic accuracy, predicts progression trajectories, identifies novel biomarkers, differentiates prodromal subtypes, and facilitates real-time clinical decisions, thereby enabling precision management across the cognitive disorder continuum (Doherty *et al*., 2023; Rehman *et al*., 2024; Onciul *et al*., 2025). Nevertheless, significant translational gaps persist in reproducibility, interpretability, and real-world generalizability, demanding resolution for clinical implementation (Martin *et al*., 2023).

Against this backdrop, this review aims to (i) synthesize advancements in AI algorithms for neuroimaging analysis; (ii) evaluate AI-powered multimodal frameworks in precision medicine for cognitive disorders; (iii) discuss persistent technical challenges and emerging solutions; and (iv) outline priority research directions. Through a critical appraisal of current applications, we seek to catalyze translational research that accelerates the clinically impactful implementation of AI in dementia diagnostics and management.

## AI algorithms and the framework applied to neuroimaging

**Figure 1:**
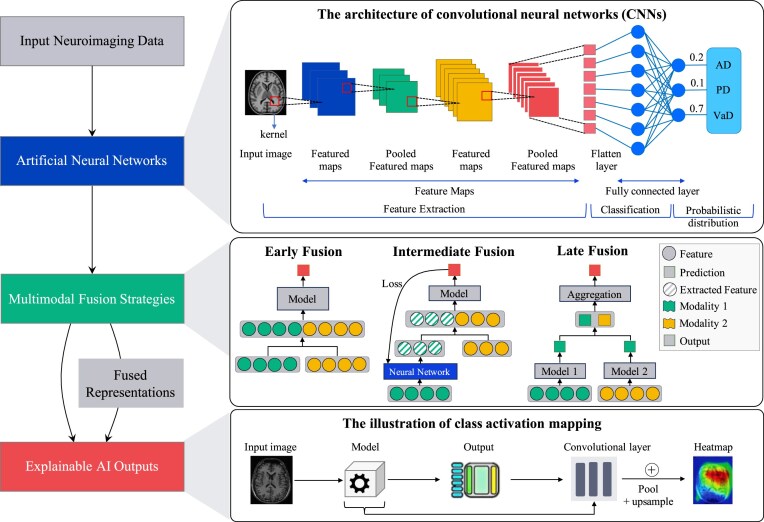
AI frameworks for neuroimaging in cognitive disorders research: key algorithms and strategies. AD, Alzheimer’s disease; PD, Parkinson’s disease; VaD, vascular dementia.

AI fundamentally utilizes computational algorithms to simulate human cognitive functions, thereby facilitating prediction, diagnosis, and prognostic assessment in individuals with cognitive disorders. Machine learning (ML), which is a broad term used to describe brain-inspired technologies, utilizes interconnected artificial neurons to achieve primitive intelligence that is analogous to that of biological systems. The operation of intelligent machines necessitates data-driven learning through training. ML algorithms identify patterns in structured historical data and extrapolate these patterns to forecast future outcomes, thus functioning within supervised (labeled outcomes) or unsupervised (unlabeled data) paradigms. Deep learning (DL), which is a specialized subset of ML, excels in processing complex unstructured data by multilayer artificial neural networks (ANNs) that hierarchically interpret information across various abstraction levels (see Fig. 1).

### Artificial neural networks

ANNs are eliciting a paradigm shift in neurodegenerative disease diagnosis, thus allowing for a transition from passive detection to active prediction. The core breakthrough of ANNs involves the implicit learning of pathological features by multilayer nonlinear transformations, thereby eliminating the explicit feature engineering that is required in traditional ML. For example, a Parkinson’s detection model based on respiratory signals can achieve noninvasive screening while constructing a continuous monitoring system for disease progression (Yang *et al*., 2022), thus indicating the evolution of ANNs from static classifiers to dynamic prognostic tools.

Among neural network architectures, convolutional neural networks (CNNs) are predominant in medical image processing because of their local receptive fields and weight-sharing mechanisms. CNNs have revolutionized structural neuroimaging through three key advances: (i) micropathological sensitivity—the ability to detect subvisual pathological signatures through hierarchical feature extraction (from local edges in shallow layers to global anatomy in deep layers)—exemplified by Korolev’s 3D-CNN reaching 80% accuracy with raw MRI data despite limited samples (Korolev *et al*., 2017); (ii) cross-modal fusion potential, as demonstrated by Mukhtar’s model, which integrates MRI, genetics, and neuropsychological data to achieve a 94% prediction accuracy (Mukhtar and Farhan, 2020); and (iii) spatiotemporal dynamics modeling, as demonstrated by the longitudinal 3D-CNN framework utilized by Yin *et al*., which has been used to quantify individualized brain aging trajectories from serial MRI scans of 2055 cognitively normal adults, thus revealing significant correlations between accelerated structural degeneration and cognitive decline to enable early AD risk assessment (Yin *et al*., 2025).

However, a critical limitation of CNNs is their heavy reliance on extensive, well-annotated datasets. Models like Korolev’s, while effective, achieve optimal performance primarily within the data distribution they were trained on, raising concerns about generalizability to heterogeneous clinical populations and differing imaging protocols (Korolev *et al*., 2017). Furthermore, while CNNs excel at identifying discriminative imaging patterns, the “black-box” nature of their deep layers often obscures the underlying neurobiological rationale for their decisions.

Graph convolutional networks (GCNs) address a fundamental neurobiological notion involving the propagation of neurodegeneration by disrupted connectomes. By modeling brains as graphs (with nodes representing regions and edges denoting structural/functional connections), GCNs can be used to identify disease-specific network fragmentation. Han’s GCN framework of resting-state functional MRI (rs-fMRI) achieved 80.3% mild cognitive impairment (MCI) diagnosis accuracy while identifying weakened connectivity in the default mode and visual networks as early biomarkers (Han *et al*., 2024); this method was complemented by Lei *et al*.’s fusion of diffusion tensor imaging (DTI) structural connectivity with rs-fMRI functional networks to attain 90.3% MCI detection accuracy (Lei *et al*., 2023). Utilizing both anatomical and network insights, a CNN–GCN hybrid architecture outperformed standalone models across 6400 scans, achieving 100% accuracy in dementia staging by feeding CNN-extracted features into GCN classifiers (Hasan and Wagler, 2024).

Building upon discriminative models such as CNNs and GCNs, generative adversarial networks (GANs) represent a paradigm shift towards addressing the fundamental challenge of data scarcity in medical AI. Rather than mapping inputs to labels, GANs learn the underlying probability distribution of the training data, enabling them to synthesize highly realistic, novel data samples (Rizvi *et al*., 2021). The integration of GANs within the ANN framework addresses pervasive multimodal data scarcity, as exemplified by the Globally & Locally Aware GAN (GLA-GAN), which synthesizes high-fidelity FDG-PET data from structural MRI, thus enabling PET-based diagnosis in resource-limited settings (Sikka *et al*., 2025). Building upon this paradigm, the Condition-Aligned Temporal Diffusion (CATD) framework pioneers electroencephalography (EEG)-to-fMRI translation through unified latent space learning by conditionally aligned blocks, which project heterogeneous EEG/fMRI data into a shared representation, whereas its dynamic time–frequency segmentation module augments the temporal resolution of fMRI by the millisecond-scale precision of EEG to capture critical neural dynamics (Yao *et al*., 2025). As validated by a 9.13% increase in brain-state prediction accuracy (69.8%) and a 4.10% increase in diagnostic precision for brain disorders (99.55%), CATD can be used to identify Parkinson’s-related abnormalities, thus establishing EEG as a scalable proxy for fMRI-quality diagnostics through cross-modal synthesis with temporal superresolution. Therefore, GANs complement discriminative networks not by making better predictions on existing data, but by expanding the very data landscape upon which all models rely.

In summary, CNNs and GCNs offer complementary perspectives (Table 1): CNNs are predominantly data-driven, excelling at identifying focal abnormalities within individual scans, whereas GCNs are mechanism-driven, modeling the systemic network disruptions that underpin disease progression. Concurrently, GANs are catalyzing a shift beyond diagnostic tasks toward solving data scarcity through realistic synthetic data generation, thereby facilitating research in resource-limited contexts. The integration of these architectures—for instance, in CNN–GCN hybrids—is consequently emerging as the most promising pathway for comprehensive and biologically grounded disease modeling.

**Table 1:** Comparative analysis of algorithms, properties, and clinical applications of CNN, GCN, and GAN in neuroimaging.

Algorithm	Strengths	Limitations	Optimal use cases
Convolutional neural networks (CNNs)	Superior spatial feature extraction from images Hierarchical learning (edges→textures→patterns) Proven high accuracy in dementia classification Extensive pretrained models available	Requires large labeled datasets Poor generalization across imaging protocols Limited biological interpretability Neglects brain network topology	Individual diagnosis from single-modality MRI Automated atrophy quantification Lesion detection in structural scans
Graph convolutional networks (GCNs)	Models brain connectome architecture Captures network-level disruption patterns Identifies circuit-based biomarkers	Dependent on accurate node/edge definition Computationally intensive preprocessing Complex hyperparameter tuning	Functional/structural connectivity analysis Network neuroscience applications
Generative adversarial networks (GANs)	Synthetic data generation Handles missing modalities Enables cross-modal translation (MRI→PET)	Training instability and mode collapse Quality control challenges for generated data Ethical concerns regarding synthetic data Validation complexity in clinical settings	Multimodal data imputation Dataset balancing for rare subtypes or prodromal stages (data scarcity)

Abbreviations: MRI, magnetic resonance imaging; PET, positron emission tomography.

### Multimodal fusion strategies

Multimodal fusion strategies (which are categorized based on integration timing into early, late, and intermediate fusion) enhance the diagnostic and prognostic robustness in individuals with cognitive disorders by integrating complementary pathological information (Ramachandram and Taylor, 2017; Tu *et al*., 2022; Odusami *et al*., 2023). Early fusion (data-level integration) combines raw or preprocessed multimodal inputs (e.g. T1-weighted MRI data with amyloid-β PET data) using dimensionality reduction techniques such as principal component analysis, independent component analysis, canonical correlation analysis, or custom image fusion algorithms (Mohammadi-Nejad *et al*., 2017). For example, Song *et al*. (2021) fused MRI and PET scans at the pixel level to generate a hybrid gray matter-PET image, which was then processed by a single diagnostic network. This approach eliminates intermodal redundancies and reduces computational complexity; however, it requires precise spatial registration of the input modalities. In another example of early fusion, Li and Fan fused baseline hippocampal MRI and longitudinal cognitive measures into a recurrent neural network (RNN) to predict the timing of MCI-to-AD dementia conversion (2019). This approach demonstrates the flexibility of data-level integration in combining heterogeneous inputs to address complex clinical challenges.

Late fusion, which is also known as decision-level aggregation, involves the training of separate models for each modality (Ramachandram *et al*., 2017). The outputs of these models are then combined using methods such as weighted averaging, Bayesian rules, or multilayer perceptron (MLP). This approach offers flexibility by accommodating asynchronous data collection, integrating heterogeneous sources (both imaging and nonimaging sources), and handling missing modalities while preserving predictive power without the need for retraining. For example, the MCI diagnosis study fused a deep learning model (trained on MRI slices) with two MLP models [for Mini-Mental State Examination (MMSE) and logical memory test scores] by majority voting, which achieved 90.9% accuracy, thereby demonstrating how late fusion is able to quantify contributions from neuroimaging and cognitive tests while mitigating bias from any single modality (Qiu *et al*., 2018). Age and sex covariates are typically integrated at this stage (Lipkova *et al*., 2022). By assigning tunable weights, late fusion quantifies the contributions of each modality, mitigates bias from dominant modalities, and reduces parameter complexity, thus making it particularly effective in data-limited scenarios.

Intermediate fusion refines feature representations through iterative optimization of modality-specific encoders within a shared latent space (Guarrasi *et al*., 2025). Generative models, particularly GANs, serve as powerful enablers of this approach. A representative example is the Joint Image Synthesis and Classification (JISCL) framework, which employs a multiscale GAN to bidirectionally synthesize MR and PET images (Z. Chen *et al*., 2025). This process extracts high-dimensional “converted features” that encapsulate cross-modal biological relationships within a unified embedding space. By integrating these synthesized features with original modality-specific inputs in a subsequent fusion module, the framework enhances diagnostic accuracy (Z. Chen *et al*., 2025)—demonstrating the potential of intermediate fusion to leverage synthetic data pathways for robust representation learning, especially in incomplete multimodal settings.

The choice of fusion strategy involves critical trade-offs: early fusion enables fine-grained feature interactions but requires complete, synchronized data; joint fusion supports flexible cross-modal integration at different abstraction levels yet demands significant data and design effort; and selection should therefore balance representational needs against real-world operational constraints such as data availability and implementation complexity.

The choice of fusion strategy is dictated by clinical context and data properties. Early fusion excels with synchronized, coregistered data by capturing fine-grained interactions, though it lacks flexibility and is less robust to noisy or incomplete data due to its direct combination of inputs (Montesinos-Lopez *et al*., 2024). Intermediate fusion supports flexible cross-modal integration at different abstraction levels yet demands significant data and design effort. In contrast, late fusion offers practical robustness for incomplete or asynchronous clinical data through modular development, albeit without modeling inter-modal relationships (Huang *et al*., 2020).

### Advancing explainable AI in neuroimaging-based dementia research

The clinical translation of AI-driven dementia diagnostics encounters significant barriers due to the inherent opacity of complex predictive models. This “black-box” problem undermines clinician trust, as an understanding of the rationale underlying patient-specific predictions (such as AD or FTD classification) is essential for treatment planning and diagnostic validation. Explainable AI (XAI) methodologies bridge this gap by elucidating algorithmic decision logic, identifying high-impact biomarkers, and generating evidence-supported justifications through techniques such as counterfactual examples (Martin *et al*., 2023). These capabilities transform AI from an inscrutable predictor into a clinically actionable decision-support tool, thus bridging the trust deficit in real-world practice.

Current XAI approaches are divided into intrinsically interpretable models and post-hoc explanation techniques (Kabir *et al*., 2025). Classical ML methods—including logistic regression, decision trees, random forests, and support vector machines (SVMs)—offer native interpretability through mathematically traceable operations. For example, Meyer *et al*. (2017) identified frontal–temporal–insular regions as critical for classifying behavioral variant FTD (bvFTD) based on SVM-derived feature weights.

In contrast, deep learning architectures necessitate post-hoc interpretation due to their hierarchical feature abstractions. Saliency mapping techniques such as Gradient-weighted Class Activation Mapping (Grad-CAM) and SHapley Additive exPlanations (SHAP) are widely used to spatially decode model activations and highlight critical brain regions (Viswan *et al*., 2024). While both generate visual explanations, their underlying mechanisms and clinical applicability differ substantially. Grad-CAM is a gradient-based technique that produces coarse, class-discriminative heatmaps by leveraging the flowing gradients of a target concept into the final convolutional layer (Selvaraju *et al*., 2017). It is particularly effective for visualizing anatomical correlates of a diagnosis—for instance, in AD detection, Grad-CAM consistently highlights expected pathological regions such as the entorhinal cortex and hippocampus, while also revealing involvement of non-traditional areas such as the optical chiasm and perivascular spaces, potentially indicative of neurovascular dysfunction in AD progression (Bloch *et al*., 2024). However, Grad-CAM explanations are model-dependent (limited to CNNs), with resolution constrained by final convolutional layer dimensions, resulting in insufficient granularity for clinical reliability (Ennab and Mcheick, 2025).

SHAP, on the other hand, is a perturbation-based method rooted in cooperative game theory. It estimates the marginal contribution of each input feature to a prediction by evaluating all possible feature combinations, ensuring properties such as efficiency, symmetry, and additivity (Lundberg and Lee, 2017). This allows SHAP to provide unified, quantitative feature importance scores across fundamentally different data types (Viswan *et al*., 2024). Its strength is particularly evident in multimodal diagnostic contexts, where it can equitably compare and rank the influence of diverse inputs—such as MRI-derived volumetric features, cognitive test scores, sociodemographic variables, and genetic markers like ApoE alleles—on a model’s classification outcome (Bloch *et al*., 2021). This capability not only clarifies which biomarkers drive individual patient classifications in heterogeneous cohorts but also supports the optimization of subject selection for balanced and representative dataset construction.

In practice, the choice between Grad-CAM and SHAP is guided by the clinical question. Grad-CAM offers an intuitive “where to look” explanation, fostering rapid visual trust in image-based models. SHAP provides a more foundational “what drove the decision” analysis, crucial for auditing complex, multimodal AI systems. These approaches should be viewed as complementary tools for model interrogation rather than as direct replacements for clinical reasoning. This integrative logic is further illustrated in applications such as prodromal Parkinson’s disease (PD) detection, where multimodal deep learning models incorporating 3D-CNNs and vision transformers have been paired with XAI (Dentamaro *et al*., 2024).

## Application of multimodal AI in cognitive impairment management

**Figure 2:**
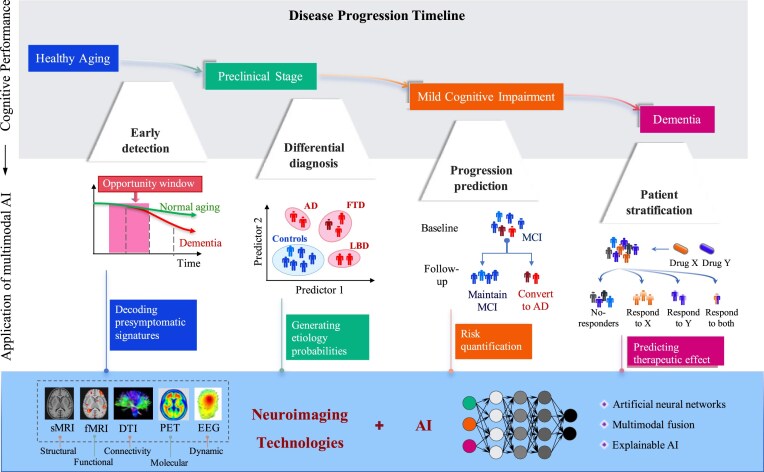
Multimodal AI neuroimaging framework for cognitive disorders. The current AI-powered framework integrates multimodal neuroimaging data [e.g. structural MRI (sMRI), functional MRI (fMRI), diffusion tensor imaging (DTI), positron emission tomography (PET), and electroencephalography (EEG) data], which are primarily used for the early detection of cognitive impairment, the differential diagnosis of dementia, the prediction of disease progression trajectories, and population risk stratification. By enabling full-spectrum intelligent diagnostics and therapeutics, this approach optimizes intervention systems for cognitive disorders.

Multimodal AI transforms cognitive impairment management by integrating complex, heterogeneous data streams to increase biomarker verification accuracy and enable timely, widely accessible diagnostics. In clinical research, AI applications leveraging multimodal neuroimaging data primarily address four critical domains (Fig. 2): (i) the differential diagnosis of dementia, (ii) the early detection of cognitive decline, (iii) the prediction of disease progression, and (iv) patient stratification for optimized therapeutic development.

### Differential diagnosis of dementia

The accurate differential diagnosis of dementia is critical for implementing targeted therapeutic interventions. While early unimodal approaches provided only partial neuropathological information, multimodal AI integration has enabled unprecedented diagnostic precision. However, critical appraisal of the literature reveals that performance gains are highly contingent on both modality selection and specific disease characteristics (see Table 2).

**Table 2:** Application of multimodal AI in differential diagnosis of dementia.

Methods	Primary application area	Modalities	Performance/results
SVM	AD/MCI/CN classification (Kohannim *et al*., 2010)	T1-MRI, FDG-PET, CSF (t-tau, p-tau, and Aβ_42_)	Unimodal (MRI alone): AD vs CN = 79.07%, MCI vs CN = 71.21%; multimodal (MRI + FDG-PET + CSF): AD vs CN = 90.70%, MCI vs CN = 75.76%
SVM	AD/CN classification (Dang *et al*., 2023)	Aβ-PET, tau-PET, FDG-PET	Unimodal (Aβ-PET/tau-PET/FDG-PET): 81%/85%/88% accuracy; multimodal (all three): 95%
MC-004 (EEG-based machine learning algorithm)	DLB vs. AD discrimination (Suzuki *et al*., 2022)	Electroencephalography	DLB vs. AD: sensitivity 72.2%, specificity 85.7%, accuracy 79.5%
ANN, SVM, and adaptive neuro-fuzzy inference systems (ANFIS)	AD vs. VaD classification (Castellazzi *et al*., 2020)	fMRI + DTI	Optimal model: ANFIS multimodal accuracy > 84%
SVM	AD vs. VaD classification (Zheng *et al*., 2019)	T1-MRI	The model achieved satisfactory AD and VaD discrimination (sensitivity: 82.65%, specificity: 87.17%, accuracy: 84.35%, AUC = 0.861)
SVM	FTD/AD/CN classification (Garcia‐Gutierrez *et al*., 2022)	Neuropsychological assessments	Accuracies > 84% for diagnosing AD, FTD, and differentiating between them; significantly reduced the number of required tests and scores
SVM	FTD vs. AD differential diagnosis (Pérez-Millan *et al*., 2024)	T1-MRI, CSF	Accuracy: 82% (MRI only) for AD vs. FTD; performance improved by adding CSF biomarkers.
3D CNN	FTD/AD/CN classification (J. Hu *et al*., 2021)	T1-MRI	Accuracy: 91.83% for multi-class classification (FTD/AD/CN).
Deep grading (Ensemble of 3D U-Nets + Multi-layer Perceptron)	FTD vs. AD detection & differential diagnosis (Nguyen *et al*., 2023)	T1-MRI	External validation accuracy: 87.1% for FTD and AD
3D VGG16-like CNN	FTD/AD/CN classification (Rogeau *et al*., 2024)	FDG-PET	Overall accuracy: 89.8%, outperformed physicians' consensus (69.5% accuracy)
Swin UNETR (a three-dimensional transformer-based architecture)	10-etiology discrimination (AD/DLB/PD/PRD/VD/FTD/MPH/ SEF/psychiatric conditions/tuberculosis) (Xue *et al*., 2024)	Multisequence MRI (T1/T2/DWI/SWI/FLAIR) + clinical data (demographics, health history)	NC/MCI/Dementia discrimination: AUC 0.94 Etiology differentiation: AUC 0.96
Logistic regression/ KNN/SVM/ naïve Bayes/Ensemble	PDD vs. DLB classification (Bougea *et al*., 2022)	Clinic + neuropsychological tests	Accuracy: KNN 91.2%, logistic regression 87.5%, SVM 84.6%, Naïve bayes 82.05%, Ensemble 89.74%
Random UnderSampling Boost/ SVM/random forest	Multi-etiology classification (AD/FTD/DLB/VaD/subjective complaints) (Tong *et al*., 2017)	T1-MRI, FLAIR, and CSF	The model achieved a high accuracy of 75.2%, which is significantly better than the results using SVM or random forest
SVM	Multi-disorder classification (AD/FTD/DLB/sPPA/CBS/ depression/subjective memory complaints) (Morin *et al*., 2020)	T1-MRI	Univariate automated volumetry software (AVS) (hippocampus): 46–71% accuracy; SVM-whole gray matter: 52–90% accuracy; SVM-AVS: 52–85% accuracy

Abbreviations: SVM, support vector machine; AD, Alzheimer’s disease; MCI, mild cognitive impairment; CN, cognitively normal; Aβ, amyloid-β; CNN, convolutional neural network; AUC, area under the curve; ANN, artificial neural network; KNN, K-nearest neighbors; CSF, cerebrospinal fluid; t-tau, total tau; p-tau, phosphorylated tau; DLB, dementia with Lewy bodies; PD, Parkinson’s disease; PDD, Parkinson’s disease dementia; FTD, frontotemporal dementia; VaD, vascular dementia; sPPA, semantic primary progressive aphasia; CBS, corticobasal syndrome; PRD, prion diseases including Creutzfeldt–Jakob disease; MPH, metabolic/paraneoplastic disorders; SEF, seizure-related encephalopathies; T1-MRI, T1-weighted magnetic resonance imaging; FLAIR, fluid-attenuated inversion recovery; PET, positron emission tomography; DTI, diffusion tensor imaging; fMRI, functional MRI.

Multimodal integration consistently outperforms unimodal approaches, yet the magnitude of improvement varies. Kohannim *et al*. (2010) demonstrated that combining MRI, FDG-PET, and cerebrospinal fluid (CSF) biomarkers yielded 5–10% accuracy gains over MRI alone. Our previous work further validated that while tau PET and FDG-PET individually outperformed amyloid-β (accuracy: 0.85/0.88 vs. 0.81), integrating all three biomarkers achieved maximal performance (0.95 accuracy; representing a 7–14% improvement) (Dang *et al*., 2023).

A critical application of multimodal AI lies in differentiating non-AD dementias, which frequently present with overlapping clinical symptoms. Growing research efforts are directed toward identifying unique digital signatures for conditions such as dementia with DLB, vascular dementia (VaD), and FTD. For example, a multicenter validation study evaluated MC-004 (an EEG-based ML algorithm) in 18 probable DLB patients and 21 probable AD patients. MC-004 achieved 72.2% sensitivity, 85.7% specificity, and 79.5% accuracy for discriminating DLB from AD, thus highlighting its potential as an accessible biomarker for enhancing DLB detection workflows (Suzuki *et al*., 2022). Additionally, Castellazzi *et al*. (2020) extracted fMRI and DTI features to compare ANN, SVM, and adaptive neuro-fuzzy inference systems (ANFIS), and they deemed ANFIS to be optimal for classifying AD versus VaD cases under multimodal feature input (accuracy > 0.84). Similarly, a radial basis function (RBF)-SVM model leveraging multiparametric structural MRI features achieved satisfactory AD/VaD discrimination (sensitivity: 82.65%, specificity: 87.17%, accuracy: 84.35%) (Zheng *et al*., 2019).

Beyond DLB and VaD, multimodal AI has shown considerable success in identifying FTD, which is characterized by frontal and temporal lobe degeneration, resulting in prominent behavioral and language deficits (Neary *et al*., 1998). Supervised ML models, particularly SVMs, have achieved high accuracy (84.5–92.6%) in distinguishing FTD from AD and healthy controls by leveraging combinations of neuropsychological assessments, PET imaging, and demographic data (Garcia‐Gutierrez *et al*., 2022; Pérez-Millan *et al*., 2024). Furthermore, DL approaches demonstrate the capability to extract complex patterns directly from raw data. Studies utilizing CNNs on MRI and FDG-PET data have reported high classification accuracies (86.0–94.6%) for differentiating FTD, AD, and healthy controls, with some models even surpassing clinical interpretation (J. Hu *et al*., 2021; Nguyen *et al*., 2023; Rogeau *et al*., 2024). These findings collectively underscore the pivotal role of multimodal AI in capturing the unique neurobiological signatures of non-AD dementias for improved differential diagnosis.

Nevertheless, the focus on a single type of dementia demonstrates limited practical relevance given the prevalence and coexistence of other etiologies. A recent large-cohort study addressed this issue by constructing a multimodal ML framework incorporating demographics, personal/family history, medication records, neuropsychological tests, functional scales, and multimodal neuroimaging to identify 10 dementia etiologies. The model attained a microaveraged AUC (area under the receiver operating characteristic curve) value of 0.94 for discriminating normal cognition, MCI, and dementia, with an AUC value of 0.96 being observed for differentiating etiologies (Xue *et al*., 2024). By generating etiology probability scores, this model quantifies pathological contributions in mixed dementia, thereby enabling clinicians to systematically prioritize evidence-based diagnostic drivers. Another prospective cohort study developed an ML classifier by using noninvasive clinical and neuropsychological tests to differentiate PD dementia (PDD) from DLB (Bougea *et al*., 2022). When trained on 78 PDD patients and 62 DLB patients with ≥3 years of diagnostic follow-up, the model demonstrated high accuracy. These studies establish an emerging paradigm: the greatest clinical value of multimodal AI lies not in pure classification accuracy, but in its ability to quantify mixed pathology and resolve complex differential diagnoses encountered in primary care settings.

Despite promising results, significant clinical translation barriers persist. A key challenge lies in the performance trade-offs inherent in complex, real-world diagnostic scenarios. For instance, the multimodal framework proposed by Tong *et al*. (2017), which employed the RUSBoost algorithm to address class imbalance, achieved an overall accuracy of 75.2% for the challenging task of five-class dementia classification. However, its balanced accuracy of 69.3% underscores the persistent difficulty in achieving equitable performance across all dementia subtypes, a common issue in clinical settings with imbalanced patient populations. Similarly, Morin *et al*. (2020) demonstrated that multimodal AI excels at differentiating diseases with distinct MRI signatures (AD/FTD/semantic dementia) but struggles with atypically presenting pathologies like DLB. These findings collectively highlight a crucial limitation: even advanced algorithms designed to handle clinical complexities like class imbalance cannot fully overcome the fundamental constraints imposed by the discriminative power of the underlying biomarkers and the heterogeneity of disease presentation.

### Early detection of cognitive impairment

Cognitive impairment exists on a spectrum ranging from normal aging to dementia, and early detection (which is crucial for timely intervention) relies on distinguishing neurodegenerative signs from typical age-related changes using clinical neuroimaging. Current clinical protocols systematically evaluate suspected dementia cases by MRI structural examinations, thereby assessing signal abnormalities and atrophy patterns (Park and Moon, 2016). For example, T1-weighted imaging can identify AD-typical medial temporal lobe and hippocampal atrophy, FTD-associated anterior temporal pole and frontal atrophy, and DLB-related subcortical atrophy. Moreover, T2-weighted or fluid-attenuated inversion recovery (FLAIR) sequences can detect vascular injury by signal changes, although these modalities may also indicate inflammatory, metabolic, toxic, or infectious processes contributing to cognitive deficits (Harper *et al*., 2014). Concurrently, FDG-PET is widely used in clinical settings for AD diagnosis and dementia subtyping, thereby revealing reduced hippocampal and posterior cingulate glucose metabolism in AD, as well as occipital hypometabolism in DLB and frontal hypometabolism in FTD (Chouliaras and O’Brien, 2023).

MRI visual rating scales (such as the medial temporal lobe atrophy rating scale) remain common in dementia diagnostics, with AUC values ranging from 0.67 to 0.97 for differential diagnosis (Harper *et al*., 2015, 2016). Automated volumetric analyses demonstrate higher precision; structural MRI atrophy maps identifying AD, DLB, or FTD patterns achieved 90% sensitivity and 84% specificity for AD, 78.7% sensitivity and 98.8% specificity for DLB, and 84.4% sensitivity and 93.8% specificity for FTD (Vemuri *et al*., 2011). In a study involving 504 participants (including AD, FTD, VaD, DLB, and control individuals), the quantification of volumetric and morphometric features based on T1 images and vascular characteristic-based FLAIR images achieved 70.6% overall accuracy, with 96% sensitivity observed for VaD, 82% sensitivity observed for controls, and 74% sensitivity observed for AD (although the sensitivity of DLB detection remained low at 32%) (Koikkalainen *et al*., 2016). While these findings establish computerized decision support as clinically viable, early cognitive impairment identification remains challenging due to frequent misattribution of initial symptoms to normal aging (Petersen *et al*., 2001; Deary *et al*., 2009).

AI is revolutionizing this paradigm by decoding presymptomatic signatures years (even decades) before clinical manifestation. Rabeh *et al*. (2016) utilized an SVM involving morphological features from the hippocampus, corpus callosum, and cortex to achieve 90.66% accuracy in early AD detection. Moreover, Ávila-Jiménez *et al*. (2024). pioneered a deep learning model using ANNs to identify subtle disease patterns in comprehensive medical records, thus significantly advancing early AD detection . Additionally, a deep learning algorithm analysing FDG-PET data from 1002 patients predicted AD diagnosis 6.3 years before clinical manifestation with 82% specificity and 100% sensitivity by detecting metabolic shifts in posterior cingulate regions (Ding *et al*., 2019). These studies demonstrated AI’s fundamental capability to detect presymptomatic changes but also revealed modality-specific limitations—structural MRI primarily captures established atrophy, while FDG-PET reflects metabolic alterations that may precede volumetric changes.

Multimodal data fusion (genetics + neuroimaging) further enhances progression prediction (Mirabnahrazam *et al*., 2022). Franzmeier *et al*. (2020) developed a biomarker-based ML algorithm trained on amyloid-β PET, structural MRI, and CSF data from 121 autosomal-dominant AD patients. Their SVM model predicted “estimated years to symptom onset” (a cognitive decline proxy); moreover, when validated in 216 sporadic AD cases, this model accurately predicted global cognitive and memory deterioration at 4 years, thus potentially reducing the clinical trial sample size requirements by 50–75%. This approach highlights a critical advantage: multimodal models capture complementary pathological processes (amyloid deposition, neurodegeneration) across different temporal stages of disease progression.

Different cognitive disorders necessitate tailored AI approaches based on their distinct pathophysiological characteristics. For PD, AI enhances early diagnostic sensitivity by detecting subtle motor and nonmotor manifestations prior to clinical diagnosis (Wu *et al*., 2023), with multimodal integration being particularly powerful. Dentamaro *et al*. (2024) achieved >90% accuracy for prodromal PD detection using a 3D-CNN architecture that synthesizes structural MRI data, cardinal motor symptoms (tremor/rigidity/bradykinesia/postural instability), and genetic risk markers, whereas Prashanth *et al*. (2016) demonstrated even higher precision (accuracy: 96.4%, sensitivity: 97.03%) by SVM, which integrates nonmotor features (including sleep behaviour disorders and olfactory loss), CSF biomarkers, and PET imaging data. These comprehensive frameworks transcend conventional diagnostic approaches by capturing early pathophysiological signatures across multiple domains (Kamran *et al*., 2021; Reddy *et al*., 2024), thereby enabling AI-guided neuroprotective interventions to be performed during the presymptomatic window to potentially slow progression. Furthermore, trajectory forecasting empowers personalized therapeutic strategies for increasingly precise PD management (Bounsall *et al*., 2023).

For vascular cognitive impairment, DL models advance VaD diagnostics by automated neuroimaging biomarker quantification. These models detect and segment critical VaD indicators [including white matter hyperintensities (WMHs), cerebral microbleeds, perivascular spaces, lacunar infarcts, and cortical atrophy] with submillimetre precision (Dong and Hayashi, 2024). DL-based WMH volumetry effectively stratifies subcortical vascular dementia severity (Joo *et al*., 2022), whereas CNN classifiers using T2-FLAIR can distinguish VaD, vascular MCI, and nonimpaired cohorts in subcortical ischemic vascular disease with 96.9% accuracy, thereby enabling the performance of early therapeutic interventions before irreversible neurological damage occurs (Yao Wang *et al*., 2019). These domain-specific advances reveal an important trend: optimal AI architecture selection is highly dependent on the specific biomarker profile and clinical manifestation pattern of each disorder.

Notably, real-world implementation faces significant diagnostic gaps, with a global pooled rate of 61.7% for undetected dementia being particularly pronounced in community settings and underserved populations (Lang *et al*., 2017). This challenge necessitates AI models capable of operating effectively with limited modalities. Emerging research demonstrates a strategic shift toward leveraging affordable technologies and cross-modal synthesis to overcome traditional diagnostic limitations. Quantitative EEG serves as a viable solution, providing a scalable screening instrument for vulnerable groups through the detection of spectral slowing and connectivity disruptions when advanced neuroimaging is inaccessible (Papaliagkas, 2025). Complementing this approach, encoder–decoder networks now synthesize cerebral blood flow measurements from structural MRI alone, delivering PET-comparable data without radioactive tracers (Hussein *et al*., 2024), while innovative GAN architectures like the Tri-Attention enhanced 3D-GAN successfully generate high-quality PET images from MRI (Sheng *et al*., 2025). Further supporting this paradigm, digital biomarkers from mobile applications enable accessible cognitive screening through ensemble AI validation (Kim *et al*., 2023), and lightweight neural networks deployed on wearable devices facilitate continuous monitoring for early MCI detection through behavioral pattern analysis (Alam *et al*., 2025). These approaches collectively highlight a transformative movement toward affordable, accessible diagnostic pathways that bypass traditional resource-intensive methods.

### Prediction of disease progression

In addition to dementia differential diagnosis, the prediction of disease progression trajectories represents a critical clinical challenge. One established methodological approach conceptualizes future state prediction as a classification task, defining specific time windows (e.g. 24 months) to distinguish patients who will reach target clinical states (including the conversion to dementia) from nonconverters (see Table 3). This paradigm has been extensively applied to predict AD progression in MCI cohorts (Veitch *et al*., 2022; Ahmadzadeh *et al*., 2023; Lee *et al*., 2024), with a recent systematic review identifying 172 dedicated studies. High-quality implementations have reported peak AUC values typically ranging from 0.80 to 0.85, thus reflecting moderate prognostic capability (Ansart *et al*., 2021).

**Table 3:** Application of multimodal AI in early detection of cognitive impairment.

AI methods	Primary application area	Modalities	Performance/results
Decision trees, random forests, support vector machines, linear regression classifiers, gradient boosting models, extreme gradient boosting	Predicting MCI-to-AD conversion (Lee *et al*., 2024)	T1-MRI, T2-FLAIR, Aβ-PET	Gradient boosting model combining all modalities achieved the best performance: sensitivity = 0.778, specificity = 0.742, AUC = 0.824
Generative adversarial network	Stratify patients at early asymptomatic stages (Skampardoni *et al*., 2024)	Genetics, CVRFs, Aβ, cognitive decline	Stratified 27 402 undiagnosed individuals into subgroups with shared variant patterns linked to genetic profiles and cognitive trajectories
Time-dependent ROC analysis	Predict MCI onset (Albert *et al*., 2018)	CSF (Aβ, phosphorylated-tau), T1-MRI, cognitive test scores, APOE genotype	Six combined measures were the most parsimonious predictors of 5-year MCI risk: AUC = 0.85, sensitivity = 0.80, specificity = 0.75. Adding cognitive domain variables significantly improved accuracy
Random forest	Predict MCI onset (Dang *et al*., 2023)	T1-MRI	The hippocampal covariance network achieved the best performance (AUC = 0.792), outperforming the default network structural covariance (AUC = 0.767), frontoparietal control network (AUC = 0.692), and hippocampal volume (AUC = 0.72)
Multi-cohort machine learning (9 ML algorithms + 8 survival analysis methods)	Predict PD-MCI and subjective cognitive decline (Loo *et al*., 2025)	Cognitive scales and Movement Disorder Society-Unified Parkinson’s Disease Rating Scale	Visuospatial ability emerged as a top predictor for PD-MCI, with better performance associated with a lower PD-MCI risk and delayed onset
Nearest neighbor classifiers	Predict 1-year disease progression rate in bvFTD (stratifying “fast progressors” vs. “slow progressors”) (Anderl‐Straub *et al*., 2021)	T1-MRI	Overall accuracy = 80% across top 50 models; specific subcortical/cortical atrophy patterns linked to rapid progression
SVM	Predict 2-year conversion from unspecific behavioral changes to bvFTD (differentiating from psychiatric/neurological mimics) (Zhutovsky *et al*., 2019)	Clinical data (behavioral symptoms), T1-MRI	Accuracy of the binary classification tasks ranged from 72 to 82%; combined clinical/MRI data outperformed single-modality approaches for early diagnosis
Longitudinal 3D-CNN	Quantify temporal pace of brain aging (Yin *et al*., 2025)	T1-MRI	The model quantified brain aging pace with an MAE = 0.16 years (7% mean error) in the test set, significantly outperforming cross-sectional models (MAE = 1.85 years, 83% error)
Graph convolutional networks	Predict dementia risk (Han *et al*., 2024)	Resting-state fMRI	Classified cognitively normal individuals from those at high risk of MCI (without clinical diagnosis) with an average accuracy = 78.8%
Elastic net regression	Predict decade-long cognitive decline and mortality (Whitman *et al*., 2025)	T1-MRI	DunedinPACNI biomarker correlated with longitudinal aging (*r* = 0.60); faster progression predicted cognitive impairment, brain atrophy, and dementia conversion

Abbreviations: AD, Alzheimer’s sisease; MCI, mild cognitive impairment; bvFTD, behavioral variant frontotemporal dementia; Aβ, amyloid-β; CNN, convolutional neural network; CVRFs, cardiovascular risk factors; AUC, area under the curve; MAE, mean absolute error; DunedinPACNI, Dunedin Pace of Aging Calculated from NeuroImaging; CSF, cerebrospinal fluid; T1-MRI, T1-weighted magnetic resonance imaging; PET, positron emission tomography; fMRI, functional MRI.

However, current multimodal prognostic models remain constrained by dementia dataset limitations, which have predominantly targeted late-stage transitions such as the MCI-to-dementia conversion, with scarce research examining individualized risk from transitions such as the normal cognition-to-MCI transition. This gap is clinically significant given the evidence that cognitively normal older adults seeking clinical evaluations experience elevated MCI risks, particularly when subtle neuropsychological declines coincide with imaging-verified atrophy (Y. Chen *et al*., 2017). Population-based analyses have confirmed that AD biomarkers (including hippocampal atrophy, amyloid imaging, and CSF measures) are poised to predict cognitive impairment onset years before the manifestation of detectable dementia symptoms (Csernansky *et al*., 2005; Moghekar *et al*., 2013; Roe *et al*., 2013). Large-scale studies are currently addressing this scenario through innovative approaches. Specifically, GANs can be used to stratify 27 402 undiagnosed individuals into subgroups with shared variant patterns linked to genetic profiles and cognitive trajectories (Skampardoni *et al*., 2024); additionally, landmark research has demonstrated that baseline CSF biomarkers, MRI measures, and APOE status can predict symptom emergence at 5-, 7-, and 10-year intervals at the individual level (Albert *et al*., 2018). Furthermore, hippocampal covariance network signatures (which reflect structural covariation patterns) outperform isolated regional measures in early MCI prediction, achieving an AUC improvement from 0.72 to 0.79 with 86% sensitivity (Dang *et al*., 2023), suggesting that network-level degeneration patterns may provide more sensitive prognostic markers than traditional regional biomarkers.

There exists a relative scarcity of research on multimodal AI for predicting progression in non-AD dementias, which stands in contrast to the well-recognized clinical need to map and anticipate their heterogeneous progression pathways. For Parkinson’s cognitive decline, a multi-cohort study leveraging ML algorithms and survival analysis methods established visuospatial dysfunction as a novel biomarker for predicting the progression of PD-MCI and subjective cognitive decline (Loo *et al*., 2025). In bvFTD, multivariate MRI analyses identified atrophy patterns in the pallidum, middle temporal gyrus, and insula as key predictors of 1-year rapid progression (“fast vs. slow progressors”) with 80% accuracy across top classification models (Anderl‐Straub *et al*., 2021). Complementarily, Zhutovsky *et al*. (2019) demonstrated that combining clinical data with voxel-wise MRI could predict 2-year conversion from nonspecific behavioral symptoms to behavioral variant FTD while differentiating from psychiatric or neurological mimics, achieving an accuracy of 72–82%. These advances collectively highlight the feasibility of individualized prognosis through modality-integrated biomarkers—from visuospatial metrics in PD to limbic-cortical degeneration signatures in bvFTD.

The velocity of brain ageing is strongly correlated with cognitive decline and neurodegenerative risk. This observation underscores the importance of brain age prediction frameworks that quantify individual deviations from normative aging trajectories by the brain-predicted age difference (Brain-PAD = predicted age − chronological age). A recent large-cohort study developed a longitudinal 3D-CNN model analysing serial MRI data to noninvasively quantify the pace of brain aging with high precision (0.16-year mean absolute error, representing an 11.5× lower error compared to cross-sectional methods), which demonstrated significant correlations with cognitive trajectories (Yin *et al*., 2025). When integrated with explainable saliency mapping, this model precisely localized neuroanatomical variations in aging rates across sex and cognitive status; specifically, females demonstrated right precentral gyrus and bilateral postcentral gyri dependence, whereas males exhibited left transverse frontopolar gyrus and right supramarginal gyrus patterns. Complementary elastic net regression modeling confirmed that single-timepoint MRI predicts decade-long cognitive decline and mortality, which has been validated across multiethnic cohorts, including Latino populations (Whitman *et al*., 2025).

Nevertheless, translating advanced progression models into clinical practice faces a fundamental challenge: the infeasibility of acquiring comprehensive multimodal data in real-world settings. The research reliance on serial MRI, longitudinal cognitive scores, and CSF biomarkers proves impractical in routine care due to cost, compliance, and operational constraints (Bernstein Sideman *et al*., 2022). To bridge this gap, future development must prioritize adaptability to incomplete and asynchronous longitudinal data through transfer learning and flexible fusion architectures. A promising approach uses encoder–decoder architectures to generate CSF biomarker proxies from structural MRI, creating non-invasive surrogates (Abrol *et al*., 2025). This approach not only circumvents invasive procedures but also identifies critical dementia-related regions with high prognostic value, enabling long-term trajectory modeling and facilitating patient triage and trial recruitment.

### Patient stratification and clinical trial optimization

The formidable challenge of developing definitive cures for dementia is underscored by the limited efficacy of current therapies in halting disease progression (Wessels *et al*., 2021)—a reality highlighted by the high failure rate in clinical trials. This therapeutic impasse extends beyond AD to other major cognitive disorders, including LBD (Agarwal *et al*., 2024), FTD (Magrath Guimet *et al*., 2022), and PDD (Szeto and Lewis, 2016), for which no disease-modifying therapies exist. Consequently, clinical management remains predominantly reliant on symptomatic treatment and multidisciplinary supportive care.

This therapeutic impasse stems from multiple factors. For example, the targeting of pathological processes such as amyloids at symptomatic stages may be too late to reverse damage, whereas clinical trial challenges include undetected phenotypic heterogeneity and failure to confirm underlying pathology in participants (Oxtoby *et al*., 2022). To address disease heterogeneity, computational models now capture distinct progression trajectories within single diagnostic categories. The Subtype and Stage Inference (SuStaIn) algorithm identifies data-driven phenotypes with unique temporal patterns from cross-sectional data (Young *et al*., 2018). In FTD, SuStaIn reveals genetic subtypes and within-genotype heterogeneity from imaging; in AD, it delineates three subtypes with characteristic neuroanatomical sequences. This fine-grained stratification significantly improves diagnostic conversion prediction over standard models, offering quantitative tools for precision neuromedicine beyond symptom-based classification.

Notably, treatment effects in clinical trials are frequently diluted by nonresponders, who ideally should be screened out, thereby prompting intensive efforts to identify probable responders (Wang *et al*., 2024). Data-driven progression models now optimize patient stratification; specifically, event-based modeling of cognitive decline can identify subgroups with severe impairment who exhibit clearer treatment responses (e.g. donepezil trial participants with amnestic MCI), whereas multimodal DL enables stratified randomization using AI-predicted cognitive decline rates as stratification indices, thereby suppressing allocation bias caused by individual variation in progression speed (Oxtoby *et al*., 2022). Complementing these computational approaches, biomarker-based enrichment strategies enhance cohort homogeneity; for example, the use of dopamine transporter SPECT excludes nondegenerative cases in PD trials (Stephenson *et al*., 2019), thus enriching idiopathic PD cohorts where reduced tracer binding predicts accelerated functional decline (UPDRS-II/III). Collectively, these innovations increase statistical power while reducing trial size, duration, and cost by ensuring that therapeutic interventions are evaluated in optimally responsive subpopulations. Furthermore, the ability of AI to utilize genetic and multiomics data also demonstrates great potential for advancing the treatment of dementia (Fang *et al*., 2022).

The personalized prediction of treatment response represents a transformative frontier in dementia care, thereby enabling clinicians to tailor interventions to individual patient profiles while optimizing clinical trial efficiency and accelerating drug development (Newby *et al*., 2023). This paradigm is exemplified by^2022^) recurrent neural network model, which was trained on anonymized UK secondary care data (NHS-CRIS) and predicts optimal cognitive-enhancing therapy (such as the use of cholinesterase inhibitors or memantine) for dementia patients based on routine clinical records. As validated across 6804 patients from two NHS trials, the algorithm identified personalized drug selections that significantly attenuated cognitive decline over two years; specifically, patients adhering to AI-recommended treatments exhibited merely a 0.60 MMSE point reduction versus a reduction of 2.80 points in the nonadherent counterparts in internal validation, with a similar protection being observed in external cohorts. This finding demonstrates the capacity of AI to distinguish between therapeutically equivalent options by forecasting individual biological responsiveness.

The promise of such precision medicine extends beyond dementia, as evidenced by cross-disease applications. The PETRUSHKA initiative employed network meta-analysis to generate antidepressant efficacy rankings incorporating patient-specific data and preference weighting (Tomlinson *et al*., 2020), whereas analogous ML approaches can successfully guide personalized therapeutics in oncology (involving chemotherapy response predictions) (Nasief *et al*., 2019; Shayesteh *et al*., 2021), epilepsy (involving antiseizure drug selection) (Croce *et al*., 2021), and psychiatric disorders (Ambrosen *et al*., 2020). These convergent advances underscore a fundamental shift from a trial-and-error prescribing process towards algorithmically driven treatment personalization, thus creating clinically actionable pathways for optimizing both symptomatic and disease-modifying interventions.

However, for clinical deployment, several key implementation barriers must be systematically addressed. Achieving seamless interoperability with diverse Electronic Health Record (EHR) systems is a fundamental challenge. Foundational models like EHR-R1, which are specifically designed for EHR analysis and have been trained on large-scale, comprehensive instruction datasets, demonstrate the direction for overcoming data heterogeneity and integrating with complex clinical notes and coding systems (Wu *et al*., 2024). Concurrently, the adoption of agentic AI assistants can help automate and simplify clinical documentation workflows, reducing the administrative burden on clinicians and facilitating the collection of structured data (Shafik, 2025). Furthermore, successful translation requires co-development with clinical workflows from the outset. This involves designing AI tools, such as the MONAI Multimodal agentic frameworks (Gupta *et al*., 2024), to support and integrate into established clinical pathways in radiology and surgery, rather than forcing post-hoc adaptations that disrupt practitioner routines.

## Challenges and potential solutions of multimodal AI applications

**Figure 3:**
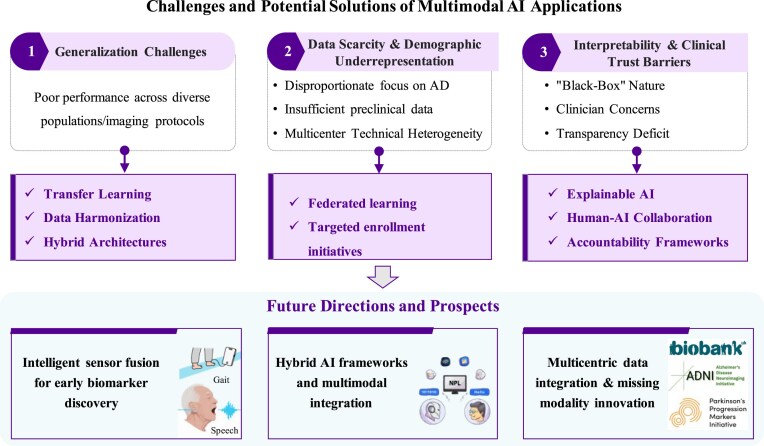
Challenges and potential solutions of multimodal AI applications in cognitive disorder research.

Multimodal AI holds transformative potential for advancing the diagnosis and management of cognitive disorders, yet critical challenges hinder its clinical translation. As depicted in Fig. 3, three interconnected barriers impede real-world deployment: (i) generalization limitations, where models fail to maintain performance across diverse populations and imaging protocols due to overfitting; (ii) data scarcity and demographic underrepresentation, exacerbating biases in non-AD and underrepresented populations; and (iii) interpretability deficits, fueling clinician distrust in “black-box” predictions. Addressing these requires synergistic technical and ethical innovations—from transfer learning and federated data harmonization to XAI frameworks—that collectively bridge the gap between algorithmic promise and clinical implementation while ensuring equitable, human-centered care.

### Generalization challenges

A critical barrier to the clinical implementation of AI decision tools is their frequent failure to generalize across heterogeneous populations and imaging protocols (Eche *et al*., 2021). Performance degradation occurs when models that are trained on institution-specific data underperform on external datasets, which is exemplified by neuroimaging classifiers maintaining 73% accuracy within training hospitals but decreasing to 55% accuracy at unseen sites (Cai *et al*., 2020). This scenario primarily stems from an issue of overfitting, where models memorize dataset-specific noise rather than generalizable biological signals, thus impairing predictive validity for new observations.

To address these challenges, three synergistic strategies show promise. (i) Transfer learning mitigates scanner and protocol variations by leveraging knowledge from large-scale source domains (Choudhary *et al*., 2020; Gholizade *et al*., 2025). For example, one study pre-trained a model on 85 721 multi-scanner MRI scans for sex classification before fine-tuning for Alzheimer’s diagnosis, achieving 90.9% external accuracy (Lu *et al*., 2022)—though this approach demands substantial computational resources and curated pre-training data to avoid negative transfer (Valverde *et al*., 2021). It is particularly suitable for extending models from well-equipped research institutions to community hospitals with differing imaging systems. (ii) Data harmonization techniques like ComBat adjust feature distributions to remove site-specific technical effects, which is crucial for multi-center studies (Gholizade *et al*., 2025). Reliable application requires adequate per-site sample sizes, detailed acquisition documentation, and standardized preprocessing to ensure feature consistency for valid statistical inference (Fortin *et al*., 2017). (iii) Hybrid architectures integrate diverse data types and learning paradigms to suppress overfitting and enhance generalizability (Schweidtmann *et al*., 2024). A CNN–SVM framework combining CNN-extracted brain features with non-imaging data achieved 0.88 accuracy and 0.95 AUC for MCI-to-AD prediction, maintaining cross-cohort performance by learning clinically meaningful representations rather than dataset-specific noise (C. Wang *et al*., 2022). Such architectures are particularly effective for building end-to-end models that natively leverage both imaging and clinical information without relying on post-hoc fusion.

### Data scarcity and demographic underrepresentation

To achieve clinical utility in resource-limited settings and effectively address complex mixed dementia pathologies, AI models must adapt to heterogeneous etiologies without relying on specialized infrastructure. However, several critical data limitations hinder this goal: (i) a disproportionate research focus on AD, with scarce multimodal datasets available for DLB, FTD, and vascular subtypes (Borchert *et al*., 2023); (ii) critically insufficient data at preclinical and prodromal stages, particularly longitudinal biomarkers, due to low screening participation, invasive sampling requirements, and reliance on caregiver-reported cognitive changes (Watson *et al*., 2014); and (iii) inconsistent feature distributions in multicenter imaging data resulting from technical heterogeneities—including differences in scanner manufacturers, magnetic field strengths (1.5T vs. 3T), and acquisition protocols—which compromise cross-site model generalizability (Kushol *et al*., 2023).

Compounding these issues is systemic demographic underrepresentation. Current datasets exhibit severe overrepresentation of Caucasian populations, despite projections that Latino and Black individuals will constitute 40% of USA Alzheimer’s cases by 2030 (Birkenbihl *et al*., 2020; Mirkin and Albensi, 2023). This disparity between training cohorts and real-world patient populations leads to performance degradation in minority groups.

Federated learning has emerged as a promising approach to address both data scarcity and privacy concerns by enabling multi-institutional collaboration while keeping data localized (Zhan *et al*., 2025). Its practical utility is illustrated in a recent study of speech-based biomarkers for cognitive impairment, which analyzed data from 2 239 participants and showed that federated learning significantly improved model performance for smaller datasets—raising balanced accuracy from 0.51 to 0.80 under class-imbalanced conditions—while maintaining robust performance (0.86 accuracy) in larger datasets (Blazquez-Folch *et al*., 2025). These findings confirm federated learning’s value as a scalable and privacy-preserving framework for developing digital biomarkers in neurodegenerative disease research, effectively mitigating data access barriers and enhancing demographic representation. Nevertheless, federated learning implementation requires participating institutions to deploy compatible deep learning frameworks and allocate substantial computational resources for local model training (Mateus *et al*., 2024).

Targeted enrollment initiatives, such as the Alzheimer’s Disease Neuroimaging Initiative (ADNI)-4 protocol, aim to enroll 50–66% of participants from underrepresented groups—and demand substantial community engagement infrastructure and culturally adapted assessment tools (Weiner *et al*., 2023). Their successful implementation hinges on establishing trust through sustained community partnerships and adapting recruitment materials to accommodate diverse literacy levels and languages. Such initiatives are particularly vital in urban medical centers serving socioeconomically diverse populations.

### Interpretability and clinical trust barriers

Although AI excels at elucidating complex patterns, its “black-box” nature creates critical trust barriers in clinical neuroscience, where decisions demonstrate profound consequences (Kiseleva *et al*., 2022). Clinicians rightfully demand explainability when AI predicts the onset of AD (regardless of the accuracy), as an understanding of the rationale directly impacts treatment planning and diagnostic validation (Xu and Shuttleworth, 2024). This transparency deficit fuels four key physician concerns: accuracy scepticism regarding real-world performance, ethical unease with respect to accountability and reliability, institutional distrust in AI procurement processes, and professional displacement anxieties (Galsgaard *et al*., 2022). As previously mentioned, emerging XAI methodologies (including attention mechanisms and saliency mapping) aim to clarify model logic by visualizing feature attribution.

Notably, studies on human–AI collaboration suggest that an appropriate level of professional skepticism may enhance clinical decision-making. Harada *et al*. (2021) demonstrated that physicians using AI differential diagnosis lists committed 14.8% fewer commission errors when maintaining professional scepticism (particularly experienced physicians). This underscores the optimal role of AI as a consultative tool rather than an autonomous diagnostician. For such systems to be effectively integrated, they must not only provide interpretable outputs but also demonstrably reduce cognitive load and decision latency, thereby supporting—rather than disrupting—clinical workflows (Olson *et al*., 2025; Pearlman *et al*., 2025). As XAI continues to bridge the interpretability gap, the development of clear accountability frameworks will be essential to ensure that these technologies augment clinicians' expertise without undermining their judgment, ultimately protecting patient welfare and reinforcing trust in AI-assisted care.

## Future directions and prospects

### Intelligent sensor fusion for early biomarker discovery

AI-enabled analysis of multimodal data streams (ranging from blood biomarkers and neuroimaging to wearable sensors) addresses computational challenges while improving biomarker identification for cognitive impairment. These advances reduce patient discomfort and diagnostic costs, thereby creating new pathways for early dementia detection. AI-driven gait analysis using inertial sensors represents a particularly promising approach. For example, Ghoraani *et al*. (2021) achieved 86% accuracy in detecting MCI and AD by SVMs analysing computerized walkway data, whereas Jeon *et al*. (2021) attained 73–74% MCI classification accuracy using accelerometer/gyroscope data during complex walking tasks. A recent validation study confirmed the reliability of such approaches, with inertial motion sensors achieving 0.961 sensitivity, 0.643 specificity, and a 0.833 AUC value for detecting cognitive impairment in community-dwelling older adults (Obuchi *et al*., 2024).

Complementary digital biomarkers derived from handwriting, speech, and environmental sensors further enable longitudinal tracking of cognitive decline (Kourtis *et al*., 2019). Passive infrared motion sensors in homes can identify MCI through movement trajectories (e.g. home-based daily walking speeds and their variability) (Akl *et al*., 2015), whereas speech analytics and handwriting dynamics provide clinically relevant indicators (Ashraf and Taati, 2015; Tóth *et al*., 2018; Angelillo *et al*., 2019).

Despite technical promise, clinical adoption faces three critical challenges. (i) The first is methodological heterogeneity in sensor placement, feature extraction, and analysis pipelines across studies, compounded by the absence of task-optimized sensor configurations (Prasanth *et al*., 2021; Niswander *et al*., 2020). Current paradigms often default to either excessive multi-sensor arrays (imposing patient burden and computational costs) or heuristic placements, ignoring that distinct pathologies manifest through domain-specific kinematic signatures. For instance, PD exhibits systemic motor deficits requiring axial trunk monitoring, whereas knee osteoarthritis demands localized joint-level sensing (Mirelman *et al*., 2019)—necessitating clinical question-tailored minimal sensor sets rather than one-size-fits-all approaches (Caramia *et al*., 2018). (ii) Environmental confounders include variability in home layouts, daily routines, and motion artifacts that degrade real-world reliability, further exacerbated by feature set inconsistency across heterogeneous devices and uncontrolled settings (Beattie *et al*., 2020; Timmons *et al*., 2025). (iii) Continuous monitoring introduces significant cybersecurity vulnerabilities and privacy risks (Timmons *et al*., 2025). Real-time data transmission without proper encryption risks interception, while long-term storage poses challenges for resource-limited institutions (Tomičić *et al*., 2022). Urgent needs include encrypted edge processing for raw data anonymization, differential privacy-compliant cloud storage, and auditable data governance to prevent breaches that erode patient trust (Mone and Shakhlo, 2023). Implementation requires robust regulatory frameworks with granular privacy safeguards, including encrypted edge processing for data anonymization, differential privacy-compliant storage solutions, and auditable data governance protocols to maintain patient trust.

### Hybrid AI frameworks and multimodal integration

Cross-modal AI integration represents a growing frontier, with one-third of recent studies combining complementary techniques for MCI detection (Hirschberg and Manning, 2015). Natural language processing (NLP) pipelines convert speech transcripts and electronic health records into analyzable vectors by linguistic embeddings, thereby enabling ML classification of cognitive states. For narrative recall tests, automated scoring employs word alignment algorithms to quantify memory retention. By combining automatic speech recognition with machine learning to analyze temporal acoustic features (e.g. speech tempo and pause patterns) in spontaneous speech during delayed recall tasks, this NLP-based method achieved a 78.8% F1 score in detecting MCI, thus demonstrating the potential for scalable community screening of early Alzheimer’s indicators (Tóth *et al*., 2018).

Computer vision (CV) synergizes with deep learning in novel applications. For example, Alzahrani *et al*. (2021). decoded cognitive status by the ML classification of eye-blinking patterns from ocular images, whereas Zolfaghari *et al*. (2022) transformed indoor locomotion patterns into visual representations for DL-based analysis . Such integrated architectures demonstrate how feature extraction from one modality (e.g. CV processing images) can optimize predictive modeling in another modality (e.g. ML/DL classification), thus expanding the analytical scope beyond unimodal approaches.

Ultimately, the clinical translation of multimodal fusion models requires rigorous validation through real-world trials, covering experimental design, data processing, modeling, testing, and safety assessment (de Hond *et al*., 2022). A major barrier remains the isolation of modality-specific processing pipelines, where structured and unstructured clinical data are often treated as separate streams rather than synergistically integrated components (Bardak and Tan, 2021; Fan *et al*., 2024). Emerging frameworks like the Medical Multimodal Pre-trained Language Model (MedM-PLM) address this through dual t networks that first extract modality-specific representations from EHR components, then employ cross-modal modules to explicitly model their bidirectional relationships (S. Liu *et al*., 2022). This approach has demonstrated enhanced EHR representations and superior performance in clinical tasks such as medication recommendation and readmission prediction.

In addition, as models incorporate more modalities, their complexity and opacity increase, raising concerns about interpretability, bias, and fairness (R. J. Chen *et al*., 2023). In response, international consensus initiatives have developed structured reporting frameworks such as the DECIDE-AI (Developmental and Exploratory Clinical Investigations of DEcision support systems driven by Artificial Intelligence) guideline (Vasey *et al*., 2022). Established through a multistakeholder Delphi process involving 123–138 experts, this 27-item checklist provides standards for early-stage clinical evaluation of AI-driven decision support systems, addressing key elements including interpretability, human-factor integration, and preliminary safety assessment.

### Multicentric data integration and missing modality innovation

Future AI development must prioritize the synthesis of multidimensional data streams—including neuroimaging, genomics, electronic health records, and real-world behavioral metrics—to enable comprehensive dementia diagnostics. The realization of this potential, however, is constrained by two critical and interrelated challenges: demographic underrepresentation and the pervasive issue of incomplete multimodal data. Large-scale initiatives such as the UK Biobank (including 100 000 participants) (Sudlow *et al*., 2015), ADNI (Mueller *et al*., 2005), and Parkinson’s Progression Markers Initiative (PPMI) (Brumm *et al*., 2023) exemplify progress in neuroimaging diversity; however, significant disparities persist in racial, socioeconomic, and rare dementia representations (e.g. early-onset AD, DLB, and FTD). Efforts like the ADNI4 expansion targeting underrepresented minorities and the formation of global consortia across Europe, Latin America, and the Asia–Pacific region are crucial steps toward building more generalizable models (Raman *et al*., 2022).

A fundamental barrier to leveraging these diverse datasets is the frequent absence of one or more data modalities in clinical practice, resulting from cost limitations, accessibility issues, and technical inconsistencies (Z. Chen *et al*., 2025). In response, the field has developed two primary, complementary strategic pathways: one focused on reconstructing missing data through generative imputation, and another centered on learning directly from available information via intermediate fusion architectures. Generative imputation methods seek to synthesize plausible versions of missing modalities, with approaches ranging from training individual models for each specific modality transition—such as generating PET from MRI using architectures like U-Net or generative adversarial networks (R. Li *et al*., 2014)—to developing unified models capable of handling multiple missing patterns through cascaded autoencoders or attention-based frameworks (Zhang *et al*., 2021). While these methods, particularly advanced ones like diffusion models (Yuanzhi Wang *et al*., 2023), can achieve high visual fidelity and help maintain performance, their effectiveness is highly dependent on the quality and completeness of the training data, and they often incur substantial computational and storage costs.

In contrast, intermediate fusion architectures circumvent the need for explicit data generation altogether (Guarrasi *et al*., 2025). Instead, they process each available modality through separate encoders and integrate their feature representations into a shared latent space within the model itself (Maheshwari *et al*., 2024). This approach demonstrates superior robustness to unpredictable missingness patterns and can maintain stable performance even when several modalities are absent simultaneously (M. Wang *et al*., 2025). By avoiding the computationally intensive step of data synthesis, intermediate fusion offers a more efficient and scalable pathway for real-world clinical deployment, particularly in resource-constrained environments where inference speed and cost are critical considerations.

The choice between these paradigms represents a strategic trade-off. Generative methods provide interpretable, data-level solutions but demand significant resources and high-quality training data, whereas intermediate fusion prioritizes computational efficiency and robustness for end-to-end diagnostic tasks. Concurrently, the advancement of modality-agnostic foundation models, pre-trained on massive multimodal datasets and subsequently fine-tuned for specific clinical scenarios (Y. Hu *et al*., 2025), offers a particularly promising pathway toward clinically deployable and generalizable AI systems.

## Conclusion

Cognitive disorders are inherently complex systemic conditions characterized by multiscale spatiotemporal interactions encompassing genetic, molecular, cellular, and neural network levels. This complexity leads to significant pathological heterogeneity and diverse clinical manifestations, thus making traditional unimodal neuroimaging techniques inadequate for comprehensive disease profiling. Crucially, the origins of cognitive decline often stem from coordinated dysregulation across structural, functional, and metabolic brain networks.

Compounding this challenge, these diseases experience prolonged preclinical progression, which is exemplified by the deposition of amyloid-beta plaques in AD, which can begin 10–20 years prior to clinical diagnosis. Current assessment tools lack the sensitivity needed to detect these early subtle changes, thus resulting in critically delayed interventions. Consequently, the elucidation of multimodal pathological cascades and the establishment of early warning systems have become imperative. Only through the cross-scale integration of neuroimaging biomarkers will it be possible to elucidate the key pathways driving cognitive decline, thereby enabling risk stratification, progression forecasting, and targeted therapeutics. The convergence of AI with multimodal neuroimaging (MRI/fMRI/PET) addresses this need by extracting nonlinear relationships from high-dimensional data to identify distributed early biomarkers, thereby ultimately bridging mechanistic insights with clinical translation.
